# Psychedelics for Cancer Pain and Associated Psychological Distress: A Narrative Review of a Potential Strategy

**DOI:** 10.1002/cam4.70586

**Published:** 2025-03-07

**Authors:** Erika Belitzky, Lis Victoria Ravani Carvalho, Melissa Taylor, Cristina Naranjo Ortiz, Laura Baum, David A. Fiellin, Maryam B. Lustberg

**Affiliations:** ^1^ Frank H. Netter M.D. School of Medicine Quinnipiac University North Haven Connecticut USA; ^2^ Medical Oncology Yale Cancer Center, Yale University New Haven Connecticut USA; ^3^ Program in Addiction Medicine Yale School of Medicine New Haven Connecticut USA

**Keywords:** cancer anxiety, cancer pain, cancer‐related distress, LSD, psilocybin, psychedelics

## Abstract

**Purpose:**

To evaluate the current level of evidence for the use of psychedelics for the management of cancer pain and associated psychological distress.

**Content:**

Pain is a common symptom of cancer and treatment. However, there are high rates of undertreatment of cancer pain due to the complex underlying biology of the condition, and potentially due to a decrease in opioid prescribing in response to the opioid epidemic. A diagnosis of cancer and cancer‐related pain can trigger high levels of psychological distress throughout cancer treatment. Cancer pain can also be exacerbated by anxiety, depression, quality of life challenges, and fear of death and dying, as well as by fear of recurrence or progression. Several pharmacologic and non‐pharmacologic approaches have been utilized to mitigate pain and symptom burden with some success. However, there remains an unmet need for better management of cancer pain and associated symptoms. Psychedelics, such as lysergic acid diethylamide (LSD), psilocybin, mescaline, and *N*,*N*‐dimethyltryptamine (DMT), are under consideration as new pharmacologic strategies for mitigating pain and the distress associated with cancer pain and associated symptom burden. Although published studies are limited, regulatory hurdles have decreased. Many clinical trials are underway to assess further the use of psychedelics and behavioral counseling for patients with cancer and comorbidities such as anxiety or depression. These studies examine both the feasibility and efficacy of psychedelics for pain and psychological distress. Early results are promising, and additional research is needed to understand efficacy and tolerability in broader cancer populations.

**Implications:**

There is an unmet need to improve pain management in patients with cancer and to mitigate psychological distress. Further research is required to understand the efficacy of psychedelics for the treatment of cancer pain and distress. Recent regulatory changes have paved the way for increased research on the clinical efficacy of psychedelics in cancer.

## 1 Introduction

Pain is a common and feared symptom of cancer and cancer treatment. Patients with cancer may experience pain due to the cancer itself or due to diagnostic and therapeutic interventions [1], and chemotherapy, surgery, hormone therapy, and radiation therapy can predispose patients to experience chronic pain syndromes [2]. Cancer‐related pain includes somatic, neuropathic, psychological, and spiritual pain. The biopsychosocial model suggests that the experience of physical symptoms, such as pain, is influenced by the dynamic interaction between physiologic, psychological, and social factors. Each individual's pain perception is unique, adding to the complexity of cancer‐related pain [3]. In advanced cancer, pain is often anticipated to be persistent throughout the course of illness and end‐of‐life care. Yet, even in those treated with curative intent, cancer pain may be ongoing for years after the initial diagnosis. Over half of the patients with cancer at any stage report some degree of pain, and more than one‐third of these patients grade their pain as moderate or severe [4]. In general, patients with more advanced tumors and those receiving multimodal therapy tend to have higher levels of pain. Increased pain is also associated with various sociodemographic factors, such as unemployment and lower income, and psychological comorbidities, such as anxiety and depression [1].

Since 1986, the World Health Organization analgesic ladder has served as the guideline for cancer‐related pain. It suggests that patients should start with a nonsteroidal anti‐inflammatory drug followed by a weak opioid if insufficient. Subsequently, a strong opioid should be used at the top of the ladder [5, 6]. A 2014 systematic review determined that the prevalence of the undertreatment of pain was 32% for articles published from 2007 to 2013, decreasing from 43% for articles published from 1987 to 2007 [7, 8]. Conversely, a living review, a systematic review that attempts to be continually updated, with papers selected from 2014 to 2020, suggested an increase to 40% of patients experiencing undertreatment of pain [9]. Cancer pain management is more complex than the ladder suggests, and the undertreatment of pain is common. Given the multitude of pain modalities, effective treatment must address various pain mechanisms.

The recent increase in undertreatment of pain may be at least partially attributed to changes in the culture and practice of opioid prescribing prompted by a reevaluation of their effectiveness and increased awareness of their risks in the setting of the opioid epidemic [10]. Multiple studies show declines in rates of opioid prescriptions in the United States for patients with Medicare and Medicaid [11, 12, 13]. In the setting of the opioid epidemic, regulations, guidelines, and insurance coverage restrictions aiming to curb inappropriate opioid prescribing have also contributed to a global decrease in opioid prescriptions, including for patients with cancer and long‐term cancer survivors [14]. Even in cancer, evidence suggests that at least one in five patients with cancer prescribed opioids is at risk of nonmedical opioid use [15]. The risk and complexity of decision‐making regarding opioids and cancer‐related pain are highest in patients with opioid use disorder, other substance use disorders, or severe mental illness who may be at high risk for opioid misuse as well as for undertreatment of their cancer pain [16]. Patients themselves may have several concerns about opioids, such as whether they are at risk for addiction or other opioid‐related harms, whether an opioid prescription signifies a transition to end‐of‐life care, and how the need for opioids is perceived by clinicians [17].

Chronic opioid use can lead to tolerance, and lack of dose adjustments can lead to inadequate pain management [9]. Traditionally, cancer‐related pain has been assessed through clinician interpretation, which may underestimate pain experience, and via questionnaires and symptom assessments in clinical trials. The reported severity of pain does not consistently match the adequacy of pain control [18]. Therefore, there is a need to find innovative and alternative pain management strategies for cancer‐related pain in this growing population of patients with cancer and cancer survivors.

Psychedelic substances have shown promise in the management of pain in a variety of chronic conditions and may provide an alternative or an adjunct to opioid use [19] [20]. The use of psychedelic substances for reducing cancer‐related pain has not yet been thoroughly studied. This review aims to evaluate the current level of evidence for the use of psychedelics as an emerging strategy in the management of pain in patients with cancer. While we aim to focus on physical pain, the experience of cancer‐related pain has a significant impact on psychological well‐being. Research to date has mostly studied the role of psychedelics in reducing distress. Distress is often coupled with the complex symptoms of cancer‐related physical pain and other psychological concerns. We use the term distress broadly to include cancer‐related anxiety, depression, quality of life issues, and existential thoughts related to fear of death and dying or fear of cancer recurrence and progression. In this article, we review the mechanism and safety of psychedelics and discuss current evidence on the use of psychedelics for cancer pain management and the reduction of cancer‐related distress, given the close interconnectedness of the two conditions.

## 2 Background

### 2.1 Emergence of Psychedelics for Pain Management

Early research in the 1960s explored the analgesic effects of LSD in comparison to opioids. One study showed that while LSD had a slower therapeutic effect, it was more effective at reducing pain than dihydromorphinone and meperidine [21]. Other early research demonstrated how LSD could work in conjunction with psychotherapy to reduce the mental and emotional distress of terminal patients with cancer [22, 23]. Grof et al. discussed that while not statistically significant, there was a definitive reduction in the need for opioid medications post‐treatment. The majority of studies evaluating the role of psychedelics on cancer‐related pain focused on populations of patients with advanced metastatic disease to improve pain and quality of life and to reduce the existential crisis related to end of life.

Non‐medical use of psychedelics and other substances in the 1960s and the concern for their potential for misuse contributed to the passage of the Controlled Substance Act of 1970 and the classification of psychedelics as a Schedule I substance with “no currently accepted medical use” [24]—this act severely limited opportunities to study these agents in clinical research. In recent years, there has been growing research interest in the potential benefits of psychedelics under close monitoring and regulatory guidelines. In June 2023, the U.S. Food and Drug Administration published its first draft of guidelines for designing and implementing clinical trials for psychedelic drugs such as psilocybin and lysergic acid diethylamide (LSD) [25]. These guidelines have paved the way for additional research on the development of safe and effective treatments with psychedelic drugs. In this article, we will focus on “classic psychedelics” which include substances such as lysergic acid diethylamide (LSD), psilocybin, mescaline, and *N*,*N*‐dimethyltryptamine (DMT) [26].

### 2.2 Mechanisms and Pharmacology

Classic psychedelics exert their effects through agonism or partial agonism of serotonin 5‐HT receptors and their subtypes [20, 24, 26]. In vitro and in vivo research demonstrates how psilocybin and psilocybin analogues have a nanomolar affinity for 5‐HT receptors, including the 5‐HT_2A_ and 5‐HT_1A_ subtypes. In separate experiments, psychedelics have induced mouse head twitches and decreased body temperature. These effects are then shown to be blocked by 5‐HT_2A_ and 5‐HT_1A_ antagonists, respectively, further supporting psychedelic activity and involvement through serotonergic pathways [27]. Serotonin receptors are widely distributed in the central nervous system and are involved in learning, memory, appetite, mood, and emotion pathways. These receptors are also found in the cardiovascular and gastrointestinal tracts and are involved in regulating vasoconstriction, heart contractility, and gastrointestinal motility [28]. This may explain some of the broader effects of psychedelics.

The role of psychedelics in pain modulation has yet to be completely understood. Pre‐clinical studies suggest that descending inhibitory serotonin pathways in the spinal cord decrease the sensitivity of dorsal horn neurons to pain fiber stimuli [19] [29]. Studies in rats suggest chronic treatment with phenylisopropylamine derivatives, which are 5‐HT2 selective agonists, regulate 5‐HT2 receptors [30, 31]. Therefore, the use of psychedelic substances may result in a decrease in 5‐HT_2A_ receptor binding sites in the brain [32]. This may occur through modulation of inflammatory gene expression and changes in neuronal network connectivity [19] [33]. Fewer serotonergic cell surface receptors can result in decreased pain transmission. In this way, classic psychedelics decrease hyperalgesia and neuropathic pain by acting through the activation of modulatory serotonergic pathways.

**FIGURE 1:**
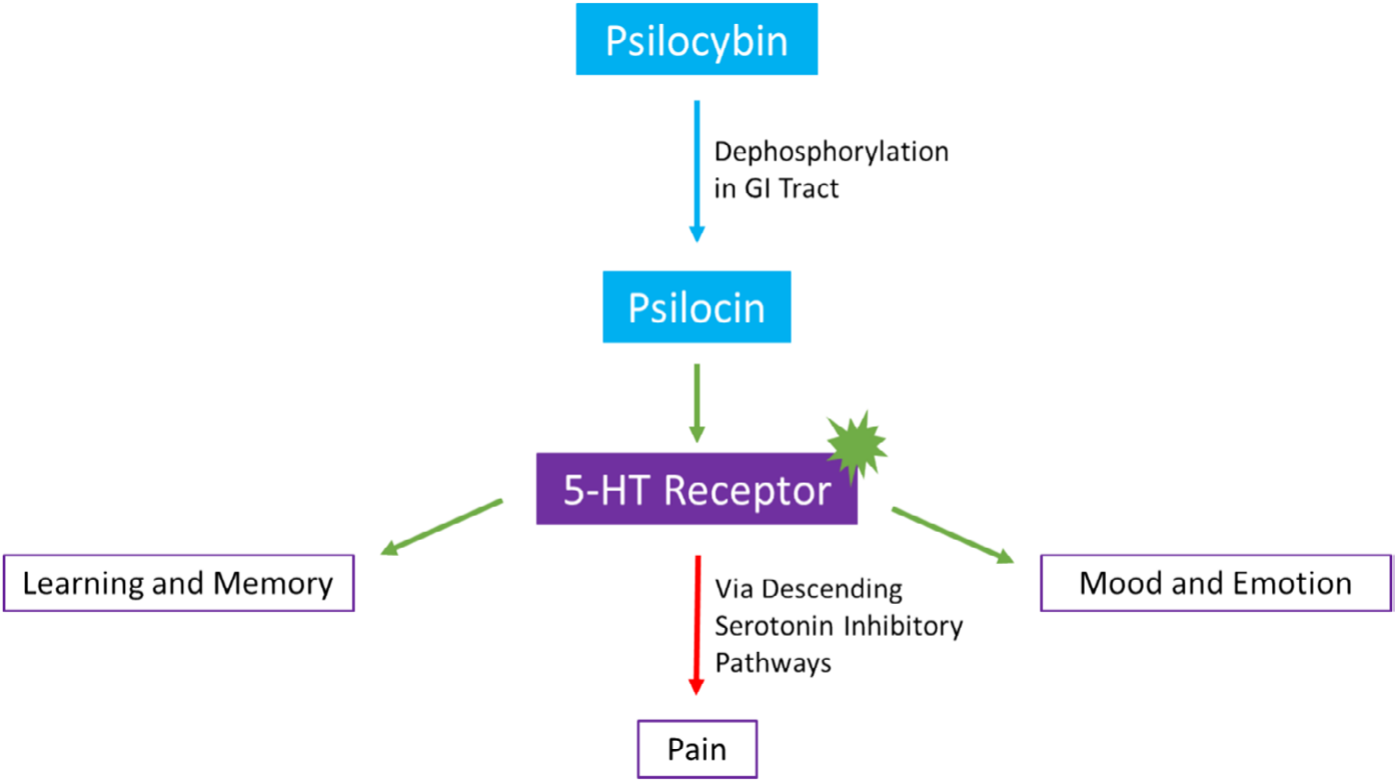
Proposed mechanism and effects of psychedelics on improving pain and distress with psilocybin.

Some psychedelics are prodrugs requiring conversion into psychoactive substances before exerting their effects. For example, psilocybin must be dephosphorylated to psilocin by the intestinal lining and liver, while DMT does not have oral bioavailability because it is eliminated by monoamine oxidase A (MAO) [34, 35]. While MAO inhibitors are less commonly used due to their side effects, the metabolism of serotonin and serotonergic hallucinogens by MAO creates the need for careful consideration of the concurrent use of psychedelics and MAO inhibitors. Figure 1 summarizes the mechanism of psychedelics, with psilocybin used as an example.

The effects of psychedelics are variable by dose, and doses for non‐medical use are typically higher than those for clinical purposes. The threshold oral dose of LSD is between 20 and 30 μg. Psilocybin is less potent, requiring oral doses between 4 and 10 mg to have an effect. These smaller therapeutic doses have mood‐altering effects, but they are less likely to have consciousness‐altering effects such as illusions compared to non‐medical use doses (75–150 μg for LSD and 10–50 mg for psilocybin). Depending on dosage, the effects can persist for approximately 6 h [36].

### 2.3 Safety and Tolerability

Although historically portrayed as dangerous, psychedelics are considered physiologically safe when administered at appropriate supervised doses. Classic hallucinogens have not been shown to result in organ damage or neuropsychological effects [37]. Symptoms such as dizziness, weakness, tremors, nausea, and blurred vision may occur. However, these are generally associated with higher doses than those used for therapeutic effect [20]. In contrast, adverse events associated with opioid analgesics, such as constipation, nausea, respiratory depression, motor and cognitive impairment, and sedation, are common, requiring extra consideration and management [5].

Safety concerns also include psychological adverse effects. Major concerns include challenging experiences, hallucinogen use disorder, and hallucinogen persistent perception disorder, which is a reoccurrence of hallucinations after the acute effects subside [38]. These risks are generally low with psychedelics that have a low potential for misuse, especially compared to opioids [39]. Data from the 2008 to 2013 National Survey on Drug Use and Health suggest psychedelic use is associated with a reduced risk of opioid dependence (defined by DSM‐IV criteria, weighted risk ratio = 0.73) and opioid misuse (weighted risk ratio = 0.60). This suggests efficacy in pain management for those with a history of substance use disorders [40].

Some studies have suggested that a single psychedelic treatment can have therapeutic benefits years after administration [41, 42]. The need for fewer doses may further improve the safety profile. Dosed under supervision, patients and family members would not have access to psychedelics, decreasing the risk of misuse. Infrequent dosing can also reduce the risk of drug interactions. While no serious adverse drug–drug interactions have been found, detailed medical screenings and further research into the interactions of psychedelics and cancer treatments are needed [43, 44]. Adverse effects can be further reduced through the use of safe and appropriate administration protocols and environment selection [19] [37].

### 2.4 Hallucinogens and Atypical Psychedelics

Classic psychedelics are a part of the broader class of drugs that result in altered states of consciousness called hallucinogens. Hallucinogens work through multiple neurotransmitter systems to produce effects on the mind and brain. Given the different mechanisms of action, these agents were excluded from the literature review. We will briefly discuss some examples of hallucinogens to provide contrast to classic psychedelics.

MDMA is an atypical psychedelic that increases the release of serotonin, dopamine, and norepinephrine as well as increases oxytocin, cortisol, and prolactin to improve empathy and emotional openness. It has a high safety profile and low addictive potential. Ketamine, an anesthetic at lower doses, can have hallucinogenic effects by acting as an NMDA‐glutamate receptor antagonist. Ketamine has a greater potential for addiction and misuse, requiring caution in dosing [45].

Cannabis works through the endocannabinoid system with receptors in the central nervous system, smooth muscle, autonomic, endocrine, and immune systems. It has therapeutic potential for chronic pain, anxiety, sleep, neurological disorders, and cancer [46] [47, 48]. However, a recent study questioned the psychedelic experience of cannabis, suggesting it may be due to expectations and not the molecular mechanism [46] [47, 48].

### 2.5 Use of Psychedelics for Physical Pain and Psychological Distress in Non‐Cancer Conditions

Results from studies looking at the role of psychedelics in the treatment of chronic pain due to other conditions may provide insight into the potential application of psychedelics in the treatment of cancer‐related pain. Chronic pain is typically defined as pain that lasts longer than the usual recovery period for a condition. Psychedelics are effective in reducing pain in a variety of conditions, such as phantom limb pain, cluster headaches, migraines, and other neuropathic conditions [49, 50, 51]. For chronic pain management, results from an online survey conducted among 250 patients who had prior experience with psychedelics, either through microdoses (small sub‐hallucinogenic doses), macrodoses (hallucinogenic doses), or both, suggested some beneficial effects in pain management for both macrodoses and microdoses [52].

Randomized control trials have shown that psilocybin, in conjunction with psychotherapy, improves anxiety and depression associated with life‐threatening illnesses [53]. LSD and *N*,*N*‐dipropyltryptamine have also been shown to be effective in reducing anxiety and depression [54]. While many studies used active placebos or other control treatments, not all trials had a control group. One open‐label pilot study found psilocybin‐assisted group therapy helpful in improving the demoralization of long‐term AIDS survivors [55]. Studies have also shown the potential of psilocybin‐assisted therapy for neurological and psychological conditions such as substance use disorder, post‐traumatic stress disorder, strokes, traumatic brain injuries, and Parkinson's disease [45]. Psychedelics, in conjunction with psychotherapy, can improve mood and quality of life.

In addition to the emotional and behavioral effects of psychedelics, research demonstrates that psychedelics may have anti‐inflammatory properties as serotonergic agonists. Pre‐clinical studies show psychedelics, such as LSD, can prevent inflammation caused by tumor necrosis factor‐alpha (TNF‐α) through activation of the 5‐HT_2A_ receptors [56, 57]. The presence of TNF‐α and other inflammatory markers indicates the potential therapeutic value of psychedelics for disorders such as asthma, atherosclerosis, rheumatoid arthritis, and irritable bowel syndrome [58]. Reduction of inflammation can, therefore, contribute to the analgesic effects of psychedelics. The purpose of the current review is to evaluate the current level of evidence for the use of psychedelics for the management of cancer pain and associated psychological distress.

## 3 Methods

**FIGURE 2:**
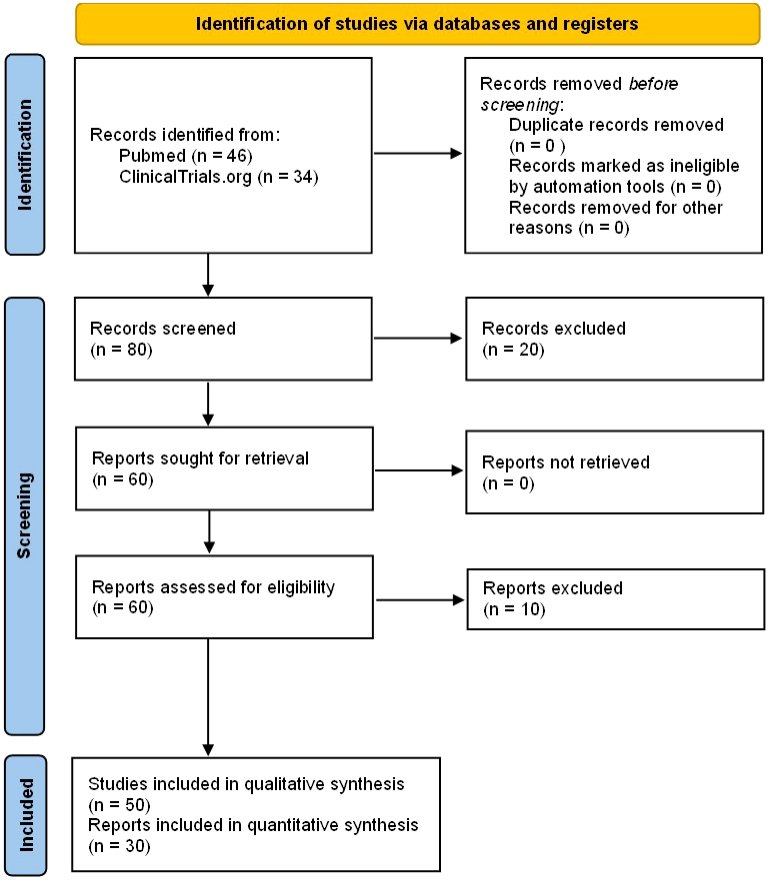
PRISMA diagram [59].

For this narrative review, we searched the PubMed database for published articles and ClinicalTrials.gov for clinical trials, using the strategies outlined in Table 1. Specifically, in the PubMed search, we focused on articles published in English. The initial search was carried out in July 2023, with subsequent monthly alerts set up until December 2023 to capture any new search results. This search yielded 46 articles, including clinical trials, basic science, and review papers, which underwent evaluation. Studies found were published between 2001 and 2023, which represent the re‐emergence of systematic studies with resulting formal legislative changes and a rise in current clinical trials. Two independent reviewers assessed relevance based on title/abstract, with a third reviewer stepping in if needed to resolve conflicts. Relevant articles then underwent full‐text review to address the research questions, though systematic quality assessment still needs to be performed. Identification and inclusion of studies are shown through a PRISMA diagram in Figure 2.

**TABLE 1:** Search strategies.

Database	Search strategy
PubMed	(((((((cancer[Title/Abstract]) OR (tumor[Title/Abstract])) OR (oncological[Title/Abstract])) AND (pain[Title/Abstract])) OR (cancer pain)) OR (“Cancer Pain”[Mesh])) AND ((((((“*N*,*N*‐Dimethyltryptamine”[Mesh]) OR “Hallucinogens”[Mesh]) OR “Psilocybin”[Mesh]) OR “Lysergic Acid Diethylamide”[Mesh]) OR “Mescaline”[Mesh]) OR (((((((((hallucinogen[Title/Abstract]) OR (psychedelic[Title/Abstract])) OR (LSD[Title/Abstract])) OR (Lysergic Acid Diethylamide[Title/Abstract])) OR (psilocybin[Title/Abstract])) OR (psilocin[Title/Abstract])) OR (N,N‐Dimethyltryptamine[Title/Abstract])) OR (DMT[Title/Abstract])) OR (Mescaline[Title/Abstract])))
ClinicalTrials.gov	Condition or Disease: “Cancer OR Cancer Pain” <br>Intervention/Treatment: “Psychedelic OR Hallucinogens and psychoactive substances OR Psilocybin OR LSD OR DMT OR Mescaline”

In July 2023, we searched ClinicalTrials.gov for clinical trials using the search strategy specified in Table 1. This yielded 30 clinical trials evaluated for relevance based on study overviews. Additionally, an additional search conducted in January 2024 identified four more studies.

## 4 Results

### 4.1 Psychedelic Use in Patients With Cancer for Physical Pain and Psychological Distress

A phase 2, fixed‐dose, open‐label study looked at the effects of psilocybin‐assisted group therapy for patients with cancer and major depressive disorder (*n* = 30). The study found NIH‐HEALS scores, a self‐administered measure of psychosocial well‐being, increased through 8 weeks following one psilocybin treatment with group sessions both in preparation and as a follow‐up [60]. Another pilot study (*n* = 12) demonstrated improvements in quality of life, spiritual well‐being, and death transcendence at 2 weeks and 6 months for patients with cancer and diagnosed with depressive disorder following psilocybin‐enhanced group psychotherapy [61]. Since group dynamics can modify the therapeutic environment, sessions were facilitated in cohorts of four participants. In both studies, fixed doses of 25 mg of psilocybin were administered with no serious adverse events.

A recent 2023 phase 2, open‐label trial was published that looked at the effects of a single dose (25 mg) of psilocybin and group therapy on depression, anxiety, pain, demoralization, and disability in patients with cancer and major depressive disorder. Results showed robust reductions in depression based on the Montgomery‐Asberg Depression Rating Scale from baseline to 8 weeks follow‐up. As exploratory outcomes, researchers looked at the effects of psilocybin on quality of life, pain, devolatilization, daily functioning, associated disability, psychosocial‐spiritual well‐being, and alterations of consciousness. There was a 37% and 26% decrease in pain based on the visual analog and the EuroQoL‐5‐dimension scales, respectively [62].

In addition to depression, psychedelics can be used to improve the anxiety associated with a cancer diagnosis. A randomized, double‐blind, crossover study compared single‐dose psilocybin (0.3 mg/kg) with a single dose of niacin (250 mg), an active control, in conjunction with psychotherapy for 29 patients with cancer. At 6.5 months of follow‐up, psilocybin was associated with anxiolytic and anti‐depressant effects and improved attitudes toward death [63]. Follow‐up assessments at 3.2 and 4.5 years demonstrated the continuation of robust decreases in suicidal ideation and loss of meaning [42]. Other long‐term follow‐up data from a longitudinal crossover study revealed that psilocybin‐assisted psychotherapy led to significant reductions in anxiety, depression, hopelessness, demoralization, and death anxiety for up to an average of 4.5 years after a single psilocybin session administered alongside psychotherapy [64].

A double‐blind randomized control trial analyzed the effects of psilocybin in 51 patients with cancer with symptoms of depression and/or anxiety. The study compared a very low (placebo‐like) dose to a high dose of psilocybin given in a living room‐like environment with no specific psychotherapy. At the 6‐month follow‐up, those in the high‐dose group showed substantial decreases in depressed mood and anxiety, with improvements in quality of life [65]. These studies indicate the potential of psychedelics alone and in conjunction with psychotherapy to improve psychosocial well‐being and quality of life. There is a need for additional randomized trials with larger sample sizes to better understand the use of psychedelics and improve generalizability.

Studies assessing psychedelics in patients with cancer for physical pain and psychological distress are case reports or small, open‐label trials. No completed trial to date has looked at cancer‐related pain as a primary outcome, and only one published study has looked at pain as a secondary, exploratory outcome. Research has focused on the potential for psychedelics to decrease cancer‐related distress and improve quality of life. One meta‐analysis, including five small trials, predominantly in cancer patients, suggested the benefit of psilocybin, LSD, and MDMA for the treatment of both anxiety and depression symptoms in patients with life‐threatening cancer [66]. This is particularly important for those with advanced, end‐stage disease experiencing existential crises. Further research is needed to improve the understanding of psychedelics in the treatment of cancer‐related pain.

### 4.2 Current Clinical Trials of Psychedelics in Patients With Cancer

Many clinical trials are underway across the United States to explore the use of psychedelics for cancer‐related pain and distress. These trials are summarized in Table 2. Of the 23 included trials, 14 use psilocybin, seven use cannabinoids, and two use MDMA, indicating the growing interest in psilocybin. All the studies are exclusively for adults. The majority are inclusive of all types of cancer, with some having additional eligibility criteria of anxiety, depression, or hopelessness related to their diagnosis. Additionally, 14 of the trials involve randomization, and 13 have some level of blinding, indicating more stringent research protocols and potential for more rigorous results as compared to older research studies.

**TABLE 2:** Summary of clinical trials involving psychedelics for cancer pain and distress.

NCT number	Study title	Phase	Intervention	Primary outcomes	Pain reduction as a primary outcome
NCT04522804 [67]	Study of psilocybin enhanced group psychotherapy in patients with cancer	EARLY_PHASE1	Psilocybin	Number of adverse events and servious adverse events for 8 months. Recruitment, enrollment, and consent for 18 months	No
NCT00957359 [68]	Psilocybin cancer anxiety study	EARLY_PHASE1	Psilocybin or niacin (active placebo)	Hospital Anxiety and Depression scores 2–4 weeks and 1 day before drug administration, and 1 day, 6 weeks, and 26 weeks post drug administration	No
NCT03661892 [69]	Pilot, syndros, decreasing use of opioids in breast cancer subjects with bone mets	EARLY_PHASE1	Syndros	Need for opiate pain mediation through use of a drug diary for 8 weeks	Yes
NCT05672342 [70]	Phytocannabinoids for the treatment of chronic chemotherapy‐induced peripheral neuropathy in breast and colon cancer survivors	EARLY_PHASE1	Cannabidiol, delta‐8‐tetrahydrocannabinol, or placebo	Difference in Functional Assessment of Cancer Therapy/Gynecologic Oncology Group (FACT/GOG)‐neurotoxicity (Ntx) subscale scores between baseline and 8 and 12 weeks of treatment for each group	No
NCT05506982 [71]	Psilocybin combined with multidisciplinary palliative care in demoralized cancer survivors with chronic pain	PHASE1	Psilocybin with psychotherapy	Incidence of treatment related adverse events occuring by day 42, patient retention in the study, number of missed events, and a survey assessing acceptability of the protocol	No
NCT05220046 [72]	Palliadelic treatment to reduce psychological distress in persons with inoperable pancreatobiliary cancer	PHASE1	Psilocybin	Recruitment rate over 18 months and retention rate over 24 months	No
NCT02675842 [73]	Investigation of cannabis for pain and inflammation in lung cancer	PHASE1	Smoked cannabis high CBD/low THC or smoked placebo cannabis low CBD/low THC	Changes in pain, sickness‐related impairment, physical symptoms, physical and emotional well being, symptoms of pain, mood, tiredness, and appetite, and quality of life over 6 weeks of treatment	Yes
NCT00302744 [74]	Effects of psilocybin in advanced‐stage cancer patients with anxiety	PHASE1/PHASE2	Psilocybin or niacin (active placebo)	Anxiety 6 months post treatment	No
NCT05272865 [75]	Pharmacokinetic and pharmacodynamic evaluation of formulations of Δ9‐THC	PHASE1/PHASE2	THC (oral formulations) or dronabinol	Pharmacokinetic assessment profile of THC formulationsm Pharmacodynamic assesment including a pain inventory and questionaire on drug effects for at least 20 days post‐chemotherapy	No
NCT05847686 [76]	Psilocybin‐assisted therapy for the treatment of cancer‐related anxiety in patients with metastatic cancer	PHASE1/PHASE2	Psilocybin with counseling	Number of instances of potentially unattended psilocybin‐related distress during psilocybin sessions	No
NCT04593563 [77]	The safety and efficacy of psilocybin in cancer patients with major depressive disorder	PHASE2	Psilocybin	Changes in depression ratings, use of pain medications, anxiety ratings, and psycho‐social spiritual healing from baseline to 8 weeks	Yes
NCT00465595 [78]	Psychopharmacology of psilocybin in cancer patients	PHASE2	Psilocybin	Changes in structured interview scores for depression and anxiety from baseline to 5 weeks and 6 months follow‐up	No
NCT00530764 [79]	A study of Sativex for pain relief in patients with advanced malignancy	PHASE2	Sativex (low, medium, or high) dose	Improvement in numberical pain rating scores from baseline to 5 weeks	Yes
NCT05584826 [80]	MDMA‐assisted therapy for adjustment disorder (AD) in dyads of patients with cancer and a concerned significant other	PHASE2	MDMA	Changes in participant scores on The Adjustment Disorder New Model and Couples Statisfaction Index from baseline to 8 weeks	No
NCT04950608 [81]	Pilot study of psilocybin‐assisted therapy for demoralization in patients receiving hospice care	PHASE2	Psilocybin with psychotherapy	Number of participants screened and enrolled per month, average time from screening to enrollment, mean number of sessions completed, proportion of planned assessments completed, duration of assesment visits, and mean score acceptability ratings. All measures are averaged over 1 year	No
NCT05947383 [82]	A phase 2, randomized, double‐blind, placebo‐controlled study to evaluate the efficacy and safety of up to two doses of psilocybin for the treatment of major depressive disorder in adults with cancer	PHASE2	Psilocybin or placebo	Change in depression scale rating from baseline to 8 weeks	No
NCT03948074 [83]	Cannabis for cancer‐related symptoms	PHASE2	Cannabis	Patients' global impression of change for overall cancer‐related symptoms 90 min after each dose of cannabis oil (1 to 6 times daily)	No
NCT00252174 [84]	MDMA‐assisted therapy in people with anxiety related to advanced stage cancer	PHASE2	MDMA with psychotherapy	Self‐reported measures of anxiety and quality of life over 3 months of active participation	No
NCT00979693 [85]	Psilocybin‐assisted psychotherapy for anxiety in people with stage IV melanoma	PHASE2	Psilocybin	Hospital Anxiety and Depression Scale scores at baseline, 1st non‐drug intro psychotherapy, day of psilocybin‐assited psychotherapy, non‐drug psychotherapy between experimental sessions, day of psilocybin‐assisted session 2, 2 weeks after second psilocybin‐assisted session	No
NCT05398484 [86]	Psilocybin therapy in advanced cancer	PHASE2|PHASE3	Psilocybin (25 mg) or niacin (100 mg) with psychotherapy	Changes in structured interview scores for anxiety from baseline to 8 weeks	No
NCT06001749 [87]	Psilocybin in cancer pain study	PHASE2	Psilocybin	Fesibility of psilocybin‐assisted therapy and acceptability of psilocybin‐assisted therapy after 3 weeks	No
NCT05629702 [88]	ARISTOCRAT: blinded trial of temozolomide +/− cannabinoids	PHASE2	Nabiximols or nabiximols‐matched placebo and temozolomide	Overall participant survival time	No
NCT06200155 [89]	Psilocybin‐assisted psychotherapy in patients with advanced cancer on maintenance therapy	PHASE2	Psilocybin or niacin	Safety and adverse events, incidence of adverse events through study completion	No

Most of the studies have endpoints that can be categorized by two themes: research feasibility and changes in participant attitudes and behaviors. Studies of feasibility are analyzing rates of recruitment, retention, and adverse events. This helps determine patient acceptance of psychedelics and the practicalities of psychedelic research. Clinical trials looking to assess the efficacy of psychedelics are often conducted through self‐reported and observer ratings and questionnaires. Most current clinical trials assess the use of psychedelics in reducing psychological distress.

Only four of the above trials use physical pain reduction as the primary outcome. One of the studies is investigating the use of dronabinol to decrease the need for opioids in breast cancer subjects with bone metastasis and is measuring the need for pain medication through a medication diary. Another study is investigating how different doses of nabiximols (cannabis extract) can provide pain relief for those with advanced malignancy by measuring changes in numerical pain ratings. The use of cannabis is also being studied for its ability to reduce pain in those with lung cancer. The only study that is currently looking at how psilocybin may affect the use of pain medications is a study looking at the safety and efficacy of psilocybin for cancer patients with major depressive disorder. Studies that have participants record symptoms and needs for pain management medications are dependent on user willingness and recollection. These journals can better capture a holistic representation of participants' responses to the intervention.

## 5 Discussion and Future Directions

Published studies and current clinical trials of psychedelics in patients with cancer suggest psychedelics have promise in reducing cancer‐related pain and psychological distress. None of the published studies and only one current trial used physical pain as a primary outcome. One of the challenges of pain research is the creation of objective measures for the subjective experience of pain. Subjective measures of pain are considered the gold standard. However, these can be limited by environmental and psychological factors, which can both overestimate and underestimate pain levels. Additionally, assessors' predispositions can affect how pain is asked about, generating reporter bias [90]. Since pain is a neurological process, it has been suggested that the pain level can be defined through biomarkers or imaging signatures resulting from the pain pathway [91]. These markers have a greater predictive value when controlled for gender and diagnosis [92]. Serum pro‐ and anti‐inflammatory cytokines have also been under investigation as they are abnormal in patients in chronic pain states [93].

Neuroimaging modalities such as structural magnetic resonance imaging (sMRI), functional MRI (fMRI), functional near‐infrared spectroscopy (fNIRS), and EEG have helped identify areas of the brain involved in acute and chronic pain. However, the predictive value of brain imaging is limited to within‐person predictions [93]. Levels of brain activity are not stimuli‐specific, and responses to non‐noxious stimuli can appear similar or identical to pain stimuli. Further research into the neurological mechanisms of pain may improve the sensitivity of brain imaging. It is thought that neuroimaging may be useful for patient stratification to predict treatment responses [94]. Functional assessments of pain, including neurotransmission, nerve transaction, and cutaneous pain assessment, aim to assess the contributions of somatosensory stimuli and peripheral innervation to pain. Evidence has demonstrated that individuals with chronic pain demonstrate pain‐modulatory imbalances. However, these functional assessments require specialized equipment and training [95]. These techniques are costly and logistically challenging. While in routine clinical use, subjective pain scales may be better able to capture the multifaceted experience of pain, objective methods have promise in research settings to minimize bias.

Many disparities already exist in the treatment of pain, with patients from underserved backgrounds receiving less adequate treatment. Racial minorities, women, individuals identifying as LGBTQ+, military veterans, those of lower socioeconomic status, and certain elderly and pediatric populations have all been identified as receiving suboptimal pain management. Black patients with cancer were less likely to be assessed for pain, and when analgesics are prescribed, they are done with less consideration for adverse effects [96]. Black patients are also more likely to be prescribed opioids and for longer compared to White patients [97]. These discrepancies result in greater dissatisfaction and mistrust of the health system. It has been proposed that complementary, non‐pharmacological therapies may address some of the discrepancies in cancer pain management. However, there are many barriers to these therapies, such as lack of insurance, negative social norms, and underrepresentation of racial and ethnic subgroups in research [98]. Research on psychedelics for cancer pain must acknowledge these disparities. Psychedelics have potential as a non‐opioid pharmacologic analgesic that can be used as part of holistic pain management.

Many published studies and current trials use psychedelics, especially psilocybin, in combination with psychotherapy. Even studies that do not have specific therapeutic guidance acknowledge the importance of the setting of psychedelic use. Psychotherapy is known to focus on improvements in physical and social functioning affecting the pain experience rather than reduction of the pain itself [99, 100]. This may make it difficult to distinguish the therapeutic benefits of psychedelics from those of psychotherapy. Future research may look to compare the benefits of psychedelics in combination with psychotherapy compared to psychotherapy alone. Several studies use psychedelics in conjunction with organized group therapy or with multiple participants receiving psychedelics simultaneously. Group dynamics may contribute to therapeutic effects and should be further explored. Research on psychedelics should also provide specifics of the nature of psychotherapy such as theoretical orientation, background of the interventionalist, number and length of sessions, and fidelity to the protocol. More work is required to understand the mechanism of how psychedelic treatment interacts with psychotherapy.

## 6 Conclusion

Pain and distress related to cancer are complex and multifactorial, including disease‐specific pain, treatment‐related effects, and psychological and spiritual distress. Classic psychedelics have a promising role in managing cancer‐related pain through their mechanism of action as serotonergic agonists and their anti‐inflammatory properties. Furthermore, their induction of an altered state of consciousness can have profound psychological effects in reducing existential distress and hopelessness related to a cancer diagnosis. While we aimed to look primarily at pain, the current body of literature on the use of psychedelics for cancer‐related pain is limited. Most studies have focused on cancer‐related distress. This distress can be related to physical pain as well as anxiety, depression, quality of life issues, and existential thoughts and fears. Several ongoing clinical trials support the potential of psychedelics and a growing interest in this field.

Further research will expand our understanding of the efficacy of psychedelics for cancer‐related pain. Findings in non‐cancer‐related chronic pain can be applied as we better understand psychedelics as an analgesic for not only somatic cancer pain but also for inflammation, neuropathic pain, spiritual pain, and the psychological or emotional states that alter the fear of our perception of pain. Adequately conducted studies that carefully evaluate the potential of psychedelics to improve the symptoms of patients with cancer will continue to expand in the near future.

## Author Contributions

**Erika Belitzky:** conceptualization (equal), data curation (equal), formal analysis (equal), investigation (equal), methodology (equal), project administration (equal), validation (equal), visualization (equal), writing – original draft (lead), writing – review and editing (equal). **Lis Victoria Ravani Carvalho:** conceptualization (equal), data curation (equal), methodology (equal), writing – review and editing (equal). **Melissa Taylor:** conceptualization (equal), writing – review and editing (equal). **Cristina Naranjo Ortiz:** methodology (equal), writing – review and editing (equal). **Laura Baum:** conceptualization (equal), methodology (equal), writing – review and editing (equal). **David A. Fiellin:** writing – review and editing (equal). **Maryam B. Lustberg:** conceptualization (equal), data curation (equal), formal analysis (equal), funding acquisition (equal), investigation (equal), methodology (equal), resources (equal), software (equal), supervision (equal), validation (equal), visualization (equal), writing – review and editing (equal).

## Conflicts of Interest

The authors declare no conflicts of interest.

## Data Availability

All data relevant to the study is included in the article.

## References

[1] M. A. Blasco , J. Cordero , and Y. Dundar , “Chronic Pain Management in Head and Neck Oncology,” Otolaryngologic Clinics of North America 53, no. 5 (2020): 865–875, 10.1016/j.otc.2020.05.015. 32684285

[2] M. M. Russo and T. Sundaramurthi , “An Overview of Cancer Pain: Epidemiology and Pathophysiology,” Seminars in Oncology Nursing 35, no. 3 (2019): 223–228. 31085106 10.1016/j.soncn.2019.04.002

[3] R. J. Gatchel , Y. B. Peng , M. L. Peters , P. N. Fuchs , and D. C. Turk , “The Biopsychosocial Approach to Chronic Pain: Scientific Advances and Future Directions,” Psychological Bulletin 133, no. 4 (2007): 581–624. 17592957 10.1037/0033-2909.133.4.581

[4] M. H. J. van den Beuken‐Everdingen , J. M. de Rijke , A. G. Kessels , H. C. Schouten , M. van Kleef , and J. Patijn , “Prevalence of Pain in Patients With Cancer: A Systematic Review of the Past 40 Years,” Annals of Oncology 18, no. 9 (2007): 1437–1449. 17355955 10.1093/annonc/mdm056

[5] R. A. Swarm , J. A. Paice , D. L. Anghelescu , et al., “Adult Cancer Pain, Version 3.2019, NCCN Clinical Practice Guidelines in Oncology,” Journal of the National Comprehensive Cancer Network 17, no. 8 (2019): 977–1007. 31390582 10.6004/jnccn.2019.0038

[6] H. Zhang , “Cancer Pain Management—New Therapies,” Current Oncology Reports 24, no. 2 (2022): 223–226. 35080737 10.1007/s11912-021-01166-z

[7] S. Deandrea , M. Montanari , L. Moja , and G. Apolone , “Prevalence of Undertreatment in Cancer Pain. A Review of Published Literature,” Annals of Oncology 19, no. 12 (2008): 1985–1991. 18632721 10.1093/annonc/mdn419 PMC2733110

[8] M. T. Greco , A. Roberto , O. Corli , et al., “Quality of Cancer Pain Management: An Update of a Systematic Review of Undertreatment of Patients With Cancer,” Journal of Clinical Oncology 32, no. 36 (2014): 4149–4154, 10.1200/JCO.2014.56.0383. 25403222

[9] A. Roberto , M. T. Greco , S. Uggeri , et al., “Living Systematic Review to Assess the Analgesic Undertreatment in Cancer Patients,” Pain Practice 22, no. 4 (2022): 487–496. 35014151 10.1111/papr.13098

[10] L. V. M. Baum , M. Kc , P. R. Soulos , et al., “Trends in New and Persistent Opioid Use in Older Adults With and Without Cancer,” JNCI: Journal of the National Cancer Institute 116, no. 2 (2024): 316–323. 37802882 10.1093/jnci/djad206 PMC13032033

[11] A. Agarwal , A. Roberts , S. B. Dusetzina , and T. J. Royce , “Changes in Opioid Prescribing Patterns Among Generalists and Oncologists for Medicare Part D Beneficiaries From 2013 to 2017,” JAMA Oncology 6, no. 8 (2020): 1271–1274. 32469405 10.1001/jamaoncol.2020.2211 PMC7260690

[12] A. C. Enzinger , K. Ghosh , N. L. Keating , D. M. Cutler , M. B. Landrum , and A. A. Wright , “US Trends in Opioid Access Among Patients With Poor Prognosis Cancer Near the End‐of‐Life,” Journal of Clinical Oncology 39, no. 26 (2021): 2948–2958. 34292766 10.1200/JCO.21.00476 PMC8425843

[13] V. Jairam , D. X. Yang , S. Pasha , et al., “Temporal Trends in Opioid Prescribing Patterns Among Oncologists in the Medicare Population,” Journal of the National Cancer Institute 113, no. 3 (2021): 274–281. 32785685 10.1093/jnci/djaa110 PMC7936059

[14] F. Chino , A. Kamal , and J. Chino , “Incidence of Opioid‐Associated Deaths in Cancer Survivors in the United States, 2006–2016: A Population Study of the Opioid Epidemic,” JAMA Oncology 6, no. 7 (2020): 1100–1102, 10.1001/jamaoncol.2020.0799. 32379275 PMC7206531

[15] S. Dalal and E. Bruera , “Pain Management for Patients With Advanced Cancer in the Opioid Epidemic Era,” American Society of Clinical Oncology Educational Book 39 (2019): 24–35. 31099619 10.1200/EDBK_100020

[16] J. V. Pergolizzi, Jr. , P. Magnusson , P. J. Christo , et al., “Opioid Therapy in Cancer Patients and Survivors at Risk of Addiction, Misuse or Complex Dependency,” Frontiers in Pain Research (Lausanne) 2 (2021): 691720. 10.3389/fpain.2021.691720 PMC8915703 35295520

[17] B. M. Scarborough and C. B. Smith , “Optimal Pain Management for Patients With Cancer in the Modern Era,” CA: A Cancer Journal for Clinicians 68, no. 3 (2018): 182–196. 29603142 10.3322/caac.21453 PMC5980731

[18] M. Thronæs , T. R. Balstad , C. Brunelli , et al., “Pain Management Index (PMI)—Does It Reflect Cancer Patients Wish for Focus on Pain?,” Supportive Care in Cancer 28, no. 4 (2020): 1675–1684. 31290020 10.1007/s00520-019-04981-0

[19] J. P. Castellanos , C. Woolley , K. A. Bruno , F. Zeidan , A. Halberstadt , and T. Furnish , “Chronic Pain and Psychedelics: A Review and Proposed Mechanism of Action,” Regional Anesthesia and Pain Medicine 45, no. 7 (2020): 486–494, 10.1136/rapm-2020-101273. 32371500

[20] N. I. Kooijman , T. Willegers , A. Reuser , et al., “Are Psychedelics the Answer to Chronic Pain: A Review of Current Literature,” Pain Practice 23, no. 4 (2023): 447–458, 10.1111/papr.13203. 36597700

[21] E. C. Kast and V. J. Collins , “Study of Lysergic Acid Diethylamide as an Analgesic Agent,” Anesthesia & Analgesia 43, no. 3 (1964): 285–291. 14169837

[22] S. Grof , L. E. Goodman , W. A. Richards , and A. A. Kurland , “LSD‐Assisted Psychotherapy in Patients With Terminal Cancer,” International Pharmacopsychiatry 8, no. 3 (1973): 129–144. 4140164 10.1159/000467984

[23] W. N. Pahnke , A. A. Kurland , L. E. Goodman , and W. A. Richards , “LSD‐Assisted Psychotherapy With Terminal Cancer Patients,” Current Psychiatric Therapies 9 (1969): 144–152. 5348915

[24] E. N. David , “Psychedelics,” Pharmacological Reviews 68, no. 2 (2016): 264. 26841800 10.1124/pr.115.011478 PMC4813425

[25] A. Grant , FDA Issues First Draft Guidance on Clinical Trials With Psychedelic Drugs (US Food and Drug Administration, 2023).

[26] H. Lowe , N. Toyang , B. Steele , et al., “Psychedelics: Alternative and Potential Therapeutic Options for Treating Mood and Anxiety Disorders,” Molecules 27, no. 8 (2022): 2520, 10.3390/molecules27082520. 35458717 PMC9025549

[27] G. A.‐O. Glatfelter , E. Pottie , J. S. Partilla , et al., “Structure‐Activity Relationships for Psilocybin, Baeocystin, Aeruginascin, and Related Analogues to Produce Pharmacological Effects in Mice,” 2022, 2575‐9108, Electronic. 10.1021/acsptsci.2c00177 PMC9667540 36407948

[28] M. Pytliak , V. Vargová , V. Mechírová , and M. Felšöci , “Serotonin Receptors – From Molecular Biology to Clinical Applications,” Physiological Research 60, no. 1 (2011): 15–25. 20945968 10.33549/physiolres.931903

[29] K. Okamoto , H. Imbe , Y. Morikawa , et al., “5‐HT2A Receptor Subtype in the Peripheral Branch of Sensory Fibers Is Involved in the Potentiation of Inflammatory Pain in Rats,” Pain 99, no. 1–2 (2002): 133–143. 12237191 10.1016/s0304-3959(02)00070-2

[30] D. J. McKenna , A. J. Nazarali , A. Himeno , and J. M. Saavedra , “Chronic Treatment With (+/−)DOI, a Psychotomimetic 5‐HT2 Agonist, Downregulates 5‐HT2 Receptors in Rat Brain,” Neuropsychopharmacology 2, no. 1 (1989): 81–87. 2803482 10.1016/0893-133x(89)90010-9

[31] M. R. Pranzatelli , “Regulation of 5‐HT2 Receptors in Rat Cortex. Studies With a Putative Selective Agonist and an Antagonist,” Biochemical Pharmacology 42, no. 5 (1991): 1099–1105. 1872895 10.1016/0006-2952(91)90294-f

[32] F. Z. Zia , M. H. Baumann , S. J. Belouin , et al., “Are Psychedelic Medicines the Reset for Chronic Pain? Preliminary Findings and Research Needs,” Neuropharmacology 233 (2023): 109528, 10.1016/j.neuropharm.2023.109528.37015315

[33] D. J. Heal , S. L. Smith , S. J. Belouin , and J. E. Henningfield , “Psychedelics: Threshold of a Therapeutic Revolution,” Neuropharmacology 236 (2023): 109610. 37247807 10.1016/j.neuropharm.2023.109610

[34] R. J. Dinis‐Oliveira , “Metabolism of Psilocybin and Psilocin: Clinical and Forensic Toxicological Relevance,” Drug Metabolism Reviews 49, no. 1 (2017): 84–91. 28074670 10.1080/03602532.2016.1278228

[35] B. Kelmendi , A. P. Kaye , C. Pittenger , and A. C. Kwan , “Psychedelics,” Current Biology 32, no. 2 (2022): R63–R67. 35077687 10.1016/j.cub.2021.12.009 PMC8830367

[36] A. Whelan and M. I. Johnson , “Lysergic Acid Diethylamide and Psilocybin for the Management of Patients With Persistent Pain: A Potential Role?,” Pain Management 8, no. 3 (2018): 217–229. 29722608 10.2217/pmt-2017-0068

[37] M. Johnson , W. Richards , and R. Griffiths , “Human Hallucinogen Research: Guidelines for Safety,” Journal of Psychopharmacology 22, no. 6 (2008): 603–620. 18593734 10.1177/0269881108093587 PMC3056407

[38] A. K. Schlag , J. Aday , I. Salam , J. C. Neill , and D. J. Nutt , “Adverse Effects of Psychedelics: From Anecdotes and Misinformation to Systematic Science,” Journal of Psychopharmacology 36, no. 3 (2022): 258–272. 35107059 10.1177/02698811211069100 PMC8905125

[39] M. W. Johnson , R. R. Griffiths , P. S. Hendricks , and J. E. Henningfield , “The Abuse Potential of Medical Psilocybin According to the 8 Factors of the Controlled Substances Act,” Neuropharmacology 142 (2018): 143–166. 29753748 10.1016/j.neuropharm.2018.05.012 PMC6791528

[40] V. D. Pisano , N. P. Putnam , H. M. Kramer , K. J. Franciotti , J. H. Halpern , and S. C. Holden , “The Association of Psychedelic Use and Opioid Use Disorders Among Illicit Users in the United States,” Journal of Psychopharmacology 31, no. 5 (2017): 606–613. 28196428 10.1177/0269881117691453

[41] M. Agrawal , S. Shnayder , H. Honstein , E. J. Emanuel , and P. M. Thambi , “Long Term Efficacy of Psilocybin in Patients With Cancer and Major Depressive Disorder (MDD),” Journal of Clinical Oncology 41, no. 16_Suppl (2023b): 12021.

[42] S. Ross , G. Agin‐Liebes , S. Lo , et al., “Acute and Sustained Reductions in Loss of Meaning and Suicidal Ideation Following Psilocybin‐Assisted Psychotherapy for Psychiatric and Existential Distress in Life‐Threatening Cancer,” ACS Pharmacology & Translational Science 4, no. 2 (2021): 553–562. 33860185 10.1021/acsptsci.1c00020 PMC8033770

[43] A. Halman , G. Kong , J. Sarris , and D. Perkins , “Drug–Drug Interactions Involving Classic Psychedelics: A Systematic Review,” Journal of Psychopharmacology 38, no. 1 (2023): 3–18. 37982394 10.1177/02698811231211219 PMC10851641

[44] C. A. MacCallum , L. A. Lo , C. A. Pistawka , and J. K. Deol , “Therapeutic Use of Psilocybin: Practical Considerations for Dosing and Administration,” Frontiers in Psychiatry 13 (2022): 1040217. 36532184 10.3389/fpsyt.2022.1040217 PMC9751063

[45] D. F. Kelly , K. Heinzerling , A. Sharma , S. Gowrinathan , K. Sergi , and R. J. Mallari , “Psychedelic‐Assisted Therapy and Psychedelic Science: A Review and Perspective on Opportunities in Neurosurgery and Neuro‐Oncology,” Neurosurgery 92, no. 4 (2023): 680–694. 36512813 10.1227/neu.0000000000002275 PMC9988324

[46] K. Cohen , A. Weizman , and A. Weinstein , “Modulatory Effects of Cannabinoids on Brain Neurotransmission,” European Journal of Neuroscience 50, no. 3 (2019): 2322–2345, 10.1111/ejn.14407. 30882962

[47] J. O. Ebbert , E. L. Scharf , and R. T. Hurt , “Medical Cannabis,” Mayo Clinic Proceedings 93, no. 12 (2018): 1842–1847. 30522595 10.1016/j.mayocp.2018.09.005

[48] C. A. Legare , W. M. Raup‐Konsavage , and K. E. Vrana , “Therapeutic Potential of Cannabis, Cannabidiol, and Cannabinoid‐Based Pharmaceuticals,” Pharmacology 107, no. 3–4 (2022): 131–149. 35093949 10.1159/000521683

[49] M. Andersson , M. Persson , and A. Kjellgren , “Psychoactive Substances as a Last Resort—A Qualitative Study of Self‐Treatment of Migraine and Cluster Headaches,” Harm Reduction Journal 14, no. 1 (2017): 60. 28870224 10.1186/s12954-017-0186-6 PMC5584001

[50] J. Fadiman and S. Korb , “Might Microdosing Psychedelics be Safe and Beneficial? An Initial Exploration,” Journal of Psychoactive Drugs 51, no. 2 (2019): 118–122. 30925850 10.1080/02791072.2019.1593561

[51] V. Ramachandran , C. Chunharas , Z. Marcus , T. Furnish , and A. Lin , “Relief From Intractable Phantom Pain by Combining Psilocybin and Mirror Visual‐Feedback (MVF),” Neurocase 24, no. 2 (2018): 105–110. 29764303 10.1080/13554794.2018.1468469

[52] V. Bonnelle , W. J. Smith , N. L. Mason , et al., “Analgesic Potential of Macrodoses and Microdoses of Classical Psychedelics in Chronic Pain Sufferers: A Population Survey,” British Journal of Pain 16, no. 6 (2022): 619–631, 10.1002/cpt.1381. 36452124 PMC9703241

[53] S. Ross , M. Agrawal , R. R. Griffiths , C. Grob , A. Berger , and J. E. Henningfield , “Psychedelic‐Assisted Psychotherapy to Treat Psychiatric and Existential Distress in Life‐Threatening Medical Illnesses and Palliative Care,” Neuropharmacology 216 (2022): 109174. 35772523 10.1016/j.neuropharm.2022.109174

[54] L. O. Maia , Y. Beaussant , and A. C. M. Garcia , “The Therapeutic Potential of Psychedelic‐Assisted Therapies for Symptom Control in Patients Diagnosed With Serious Illness: A Systematic Review,” Journal of Pain and Symptom Management 63, no. 6 (2022): e725‐e738. 35157985 10.1016/j.jpainsymman.2022.01.024

[55] B. T. Anderson , A. Danforth , P. R. Daroff , et al., “Psilocybin‐Assisted Group Therapy for Demoralized Older Long‐Term AIDS Survivor Men: An Open‐Label Safety and Feasibility Pilot Study,” EClinicalMedicine 27 (2020): 100538. 33150319 10.1016/j.eclinm.2020.100538 PMC7599297

[56] F. Nau, Jr. , B. Yu , D. Martin , and C. D. Nichols , “Serotonin 5‐HT2A Receptor Activation Blocks TNF‐α Mediated Inflammation In Vivo,” PLoS One 8, no. 10 (2013): e75426. 24098382 10.1371/journal.pone.0075426 PMC3788795

[57] D. E. Nichols , M. W. Johnson , and C. D. Nichols , “Psychedelics as Medicines: An Emerging New Paradigm,” Clinical Pharmacology & Therapeutics 101, no. 2 (2017): 209–219, 10.1002/cpt.557. 28019026

[58] T. W. Flanagan and C. D. Nichols , “Psychedelics as Anti‐Inflammatory Agents,” International Review of Psychiatry 30, no. 4 (2018): 363–375. 30102081 10.1080/09540261.2018.1481827

[59] M. J. Page , J. E. McKenzie , P. M. Bossuyt , et al., “The PRISMA 2020 Statement: An Updated Guideline for Reporting Systematic Reviews,” BMJ 372 (2021): n71. 33782057 10.1136/bmj.n71 PMC8005924

[60] S. Shnayder , R. Ameli , N. Sinaii , A. Berger , and M. Agrawal , “Psilocybin‐Assisted Therapy Improves Psycho‐Social‐Spiritual Well‐Being in Cancer Patients,” Journal of Affective Disorders 323 (2023): 592–597. 36513161 10.1016/j.jad.2022.11.046 PMC9884542

[61] B. R. Lewis , E. L. Garland , K. Byrne , et al., “HOPE: A Pilot Study of Psilocybin Enhanced Group Psychotherapy in Patients With Cancer,” Journal of Pain and Symptom Management 66, no. 3 (2023): 258–269, 10.1016/j.jpainsymman.2023.06.006. 37302533

[62] M. Agrawal , W. Richards , Y. Beaussant , et al., “Psilocybin‐Assisted Group Therapy in Patients With Cancer Diagnosed With a Major Depressive Disorder,” Cancer 130, no. 7 (2024): 1137–1146. 38105655 10.1002/cncr.35010

[63] S. Ross , A. Bossis , J. Guss , et al., “Rapid and Sustained Symptom Reduction Following Psilocybin Treatment for Anxiety and Depression in Patients With Life‐Threatening Cancer: A Randomized Controlled Trial,” Journal of Psychopharmacology 30, no. 12 (2016): 1165–1180, 10.1177/0269881116675512. 27909164 PMC5367551

[64] G. I. Agin‐Liebes , T. Malone , M. M. Yalch , et al., “Long‐Term Follow‐Up of Psilocybin‐Assisted Psychotherapy for Psychiatric and Existential Distress in Patients With Life‐Threatening Cancer,” Journal of Psychopharmacology 34, no. 2 (2020): 155–166. 31916890 10.1177/0269881119897615

[65] R. R. Griffiths , M. W. Johnson , M. A. Carducci , et al., “Psilocybin Produces Substantial and Sustained Decreases in Depression and Anxiety in Patients With Life‐Threatening Cancer: A Randomized Double‐Blind Trial,” Journal of Psychopharmacology 30, no. 12 (2016): 1181–1197, 10.1177/0269881116675513. 27909165 PMC5367557

[66] D. Sicignano , K. Snow‐Caroti , A. V. Hernandez , and C. M. White , “The Impact of Psychedelic Drugs on Anxiety and Depression in Advanced Cancer or Other Life‐Threatening Disease: A Systematic Review With Meta‐Analysis,” American Journal of Clinical Oncology 46, no. 6 (2023): 236–245. 36907889 10.1097/COC.0000000000000998

[67] U.S. National Library of Medicine , “Study of Psilocybin Enhanced Group Psychotherapy in Patients With Cancer (NCT04522804),” 2023, https://beta.clinicaltrials.gov/study/NCT04522804.

[68] U.S. National Library of Medicine , “Psilocybin Cancer Anxiety Study (NCT00957359),” 2023, https://beta.clinicaltrials.gov/study/NCT00957359.

[69] U.S. National Library of Medicine , “Pilot, Syndros, Decreasing Use of Opioids in Breast Cancer Subjects With Bone Mets (NCT03661892),” 2023, https://beta.clinicaltrials.gov/study/NCT03661892.

[70] U.S. National Library of Medicine , “Phytocannabinoids for the Treatment of Chronic Chemotherapy‐Induced Peripheral Neuropathy in Breast and Colon Cancer Survivors (NCT05672342),” 2023, https://beta.clinicaltrials.gov/study/NCT05672342.

[71] U.S. National Library of Medicine , “Psilocybin Combined With Multidisciplinary Palliative Care in Demoralized Cancer Survivors With Chronic Pain (NCT05506982),” 2023, https://beta.clinicaltrials.gov/study/NCT05506982.

[72] U.S. National Library of Medicine , “Palliadelic Treatment to Reduce Psychological Distress in Persons With Inoperable Pancreatobiliary Cancer (NCT05220046),” 2023, https://beta.clinicaltrials.gov/study/NCT05220046.

[73] U.S. National Library of Medicine , “Investigation of Cannabis for Pain and Inflammation in Lung Cancer (NCT02675842),” 2023, https://beta.clinicaltrials.gov/study/NCT02675842.

[74] U.S. National Library of Medicine , “Effects of Psilocybin in Advanced‐Stage Cancer Patients With Anxiety (NCT00302744),” 2023, https://beta.clinicaltrials.gov/study/NCT00302744.

[75] U.S. National Library of Medicine , “Pharmacokinetic and Pharmacodynamic Evaluation of Formulations of Δ9‐THC (NCT05272865),” 2023, https://beta.clinicaltrials.gov/study/NCT05272865.

[76] U.S. National Library of Medicine , “Psilocybin‐Assisted Therapy for the Treatment of Cancer‐Related Anxiety in Patients With Metastatic Cancer (NCT05847686),” 2023, https://beta.clinicaltrials.gov/study/NCT05847686.

[77] U.S. National Library of Medicine , “The Safety and Efficacy of Psilocybin in Cancer Patients With Major Depressive Disorder (NCT04593563),” 2023, https://beta.clinicaltrials.gov/study/NCT04593563.

[78] U.S. National Library of Medicine , “Psychopharmacology of Psilocybin in Cancer Patients (NCT00465595),” 2023, https://beta.clinicaltrials.gov/study/NCT00465595.

[79] U.S. National Library of Medicine , “A Study of Sativex for Pain Relief in Patients With Advanced Malignancy. (NCT00530764),” 2023, https://beta.clinicaltrials.gov/study/NCT00530764.

[80] U.S. National Library of Medicine , “MDMA‐Assisted Therapy for Adjustment Disorder (AD) in Dyads of Patients With Cancer and a Concerned Significant Other (NCT05584826),” 2023, https://beta.clinicaltrials.gov/study/NCT05584826.

[81] U.S. National Library of Medicine , “Pilot Study of Psilocybin‐Assisted Therapy for Demoralization in Patients Receiving Hospice Care (NCT04950608),” 2023, https://beta.clinicaltrials.gov/study/NCT04950608.

[82] U.S. National Library of Medicine , “A Phase 2, Randomized, Double‐Blind, Placebo‐Controlled Study to Evaluate the Efficacy and Safety of up to Two Doses of Psilocybin for the Treatment of Major Depressive Disorder in Adults With Cancer (NCT05947383),” 2023, https://beta.clinicaltrials.gov/study/NCT05947383.

[83] U.S. National Library of Medicine , “Cannabis for Cancer‐Related Symptoms (NCT03948074),” 2023, https://beta.clinicaltrials.gov/study/NCT03948074.

[84] U.S. National Library of Medicine , “MDMA‐Assisted Therapy in People With Anxiety Related to Advanced Stage Cancer (NCT00252174),” 2023, https://beta.clinicaltrials.gov/study/NCT00252174.

[85] U.S. National Library of Medicine , “Psilocybin‐Assisted Psychotherapy for Anxiety in People With Stage IV Melanoma (NCT00979693),” 2023, https://beta.clinicaltrials.gov/study/NCT00979693.

[86] U.S. National Library of Medicine , “Psilocybin Therapy in Advanced Cancer (NCT05398484),” 2023, https://beta.clinicaltrials.gov/study/NCT05398484.

[87] U.S. National Library of Medicine , “Psilocybin in Cancer Pain Study (NCT06001749),” 2024, https://clinicaltrials.gov/study/NCT06001749.

[88] U.S. National Library of Medicine , “ARISTOCRAT: Blinded Trial of Temozolomide +/− Cannabinoids (NCT05629702),” 2024, https://clinicaltrials.gov/study/NCT05629702.

[89] U.S. National Library of Medicine , “Psilocybin‐Assisted Psychotherapy in Patients With Advanced Cancer on Maintenance Therapy (NCT06200155),” 2024, https://clinicaltrials.gov/study/NCT06200155.

[90] X. Xu and Y. Huang , “Objective Pain Assessment: A Key for the Management of Chronic Pain,” F1000Research 9 (2020): F1000 Faculty Rev‐35. 10.12688/f1000research.20441.1 PMC6979466 32047606

[91] D. Borsook , L. Becerra , and R. Hargreaves , “Biomarkers for Chronic Pain and Analgesia. Part 1: The Need, Reality, Challenges, and Solutions,” Discovery Medicine 11, no. 58 (2011): 197–207. 21447279

[92] A. B. Niculescu , H. Le‐Niculescu , D. F. Levey , et al., “Towards Precision Medicine for Pain: Diagnostic Biomarkers and Repurposed Drugs,” Molecular Psychiatry 24, no. 4 (2019): 501–522. 30755720 10.1038/s41380-018-0345-5 PMC6477790

[93] S. Eldabe , I. Obara , C. Panwar , and D. Caraway , “Biomarkers for Chronic Pain: Significance and Summary of Recent Advances,” Pain Research and Management 2022 (2022): 1940906. 36385904 10.1155/2022/1940906 PMC9663208

[94] A. Mouraux and G. D. Iannetti , “The Search for Pain Biomarkers in the Human Brain,” Brain 141, no. 12 (2018): 3290–3307. 30462175 10.1093/brain/awy281 PMC6262221

[95] R. B. Fillingim , J. D. Loeser , R. Baron , and R. R. Edwards , “Assessment of Chronic Pain: Domains, Methods, and Mechanisms,” Journal of Pain 17, no. 9, Supplement (2016): T10–T20. 27586827 10.1016/j.jpain.2015.08.010 PMC5010652

[96] L. H. Nguyen , J. E. Dawson , M. Brooks , J. S. Khan , and N. Telusca , “Disparities in Pain Management,” Anesthesiology Clinics 41, no. 2 (2023): 471–488. 37245951 10.1016/j.anclin.2023.03.008

[97] S. J. Kim , R. P. Retnam , A. L. Sutton , M. C. Edmonds , D. Bandyopadhyay , and V. B. Sheppard , “Racial Disparities in Opioid Prescription and Pain Management Among Breast Cancer Survivors,” Cancer Medicine 12, no. 9 (2023): 10851–10864. 36916310 10.1002/cam4.5755 PMC10225217

[98] K. T. Liou , R. Ashare , B. Worster , et al., “SIO‐ASCO Guideline on Integrative Medicine for Cancer Pain Management: Implications for Racial and Ethnic Pain Disparities,” JNCI Cancer Spectrum 7, no. 4 (2023): pkad042. 37307074 10.1093/jncics/pkad042 PMC10336300

[99] A. Ruano , F. García‐Torres , M. Gálvez‐Lara , and J. A. Moriana , “Psychological and Non‐Pharmacologic Treatments for Pain in Cancer Patients: A Systematic Review and Meta‐Analysis,” Journal of Pain and Symptom Management 63, no. 5 (2022): e505–e520. 34952171 10.1016/j.jpainsymman.2021.12.021

[100] J. A. Sturgeon , “Psychological Therapies for the Management of Chronic Pain,” Psychology Research and Behavior Management 7 (2014): 115–124. 24748826 10.2147/PRBM.S44762 PMC3986332

